# The Regulation of Ruminal Short-Chain Fatty Acids on the Functions of Rumen Barriers

**DOI:** 10.3389/fphys.2019.01305

**Published:** 2019-10-25

**Authors:** Hong Shen, Zhihui Xu, Zanming Shen, Zhongyan Lu

**Affiliations:** ^1^College of Life Sciences, Nanjing Agricultural University, Nanjing, China; ^2^Bioinformatics Center, Nanjing Agricultural University, Nanjing, China; ^3^Key Lab of Animal Physiology and Biochemistry, College of Veterinary Medicine, Nanjing Agricultural University, Nanjing, China

**Keywords:** epimural microbiota, rumen barrier, short-chain fatty acids, epithelium physiology, microbe-host interactions

## Abstract

The rumen barriers, constituted by the microbial, physical and immune barrier, prevent the transmission of pathogens and toxins to the host tissue in the maintenance of host-microbe homeostasis. Ruminal short-chain fatty acids (SCFAs), which are the important signaling molecules derived from the rumen microbiota, regulate a variety of physiological functions of the rumen. So far, how the ruminal SCFAs regulate the function of rumen barriers is unclear. By the combined methods of transcriptome sequencing, 16S *rRNA* gene sequencing, and metagenome shotgun sequencing, we have investigated the regulatory effects of ruminal SCFAs on the functions of rumen barriers, by determining the composition and functions of epimural microbiota and on the structure and immunity of the rumen epithelium in goats receiving a 10% (LC group), 35% (MC group), or 65% concentrate diet (HC group). We found that, when the dietary concentrate shifted from 10 to 35%, the increase of total SCFA is associated with the diversification of epimural microbiota and the diversity of its gene pool. Within the microbial community, the relative abundance of genera *Sphingobium*, *Acinetobacter*, and *Streptococcus* increase mostly. Meantime, the signals on pathways concerning the mechanical connections and growth homeostasis in the rumen epithelium were upregulated. Under these conditions, the responses of immune components in the rumen epithelium decrease. However, when the dietary concentrate shifted from 35 to 65%, the increase of acetate and reduction of pH decrease the diversity of epimural microbiota and the diversity of its gene pool. Within the microbial community, the relative abundance of genera *Sphingobium*, *Acinetobacter*, and *Streptococcus* significantly decrease. Concomitantly, the signals on pathways concerning the cell growth and tight junction disruption were upregulated, while the signals on pathways concerning paracellular permeability were downregulated. Under these conditions, the signals on the pathways relating to the immune components increase. Our data thus indicates that diet-SCFA axis maintains the host-microbe homeostasis via promoting the diversification of epimural microbiota and maintaining the integrity of rumen epithelium in healthy animals, while via enhancing the activities of immune barrier in animal with lower rumen pH.

## Introduction

The rumen is the most important site for digestion in ruminant animals. On the one hand, it provides the space and nutrients for microbes to live within the rumen. On the other hand, the ruminal microbes ferment plant materials into short-chain fatty acids (SCFAs) that regulate a variety of physiological functions of the rumen (Li et al., 2016). During long-term evolution, various strategies have been developed by the animals and microbes to control their relationships. The central strategy utilized by them to maintain such homeostatic relationships is to construct barriers and, therefore, to protect the ecological niche of the commensals, limit the colonization of pathogens, and clearance the invaded microbes in the intestinal epithelium (Belkaid and Hand, 2014).

The barriers of rumen are constituted by three parts: (1) the microbial barrier, which is composed primarily of the microbes attaching to the surface of the stratified squamous epithelium (termed the epimural microbes). The commensals within epimural microbiome could inhibit the colonization of pathogens in the epithelium via the competition of shared nutritions, secretion of antimicrobial components (e.g., bacteriocins, microcins, and colicins), and alteration of environmental conditions required for the growth of pathogens (Ohland and Jobin, 2015). For example, *Bifidobacterium* inhibited the colonization of pathogenic *Escherichia coli* by decreasing the acetate concentration (Fukuda et al., 2011). Moreover, the commensals could inhibit pathogen virulence by suppressing the expression of the virulence gene. For example, *Bacteroides thetaiotaomicron* modulates the expression of the virulence factor *ler* in pathogenic *E. coli* by generating the fucose, a metabolite of host mucin (Pacheco et al., 2012); (2) the physical barrier, which is composed of the epithelial cells and the mechanical connections between the epithelial cells. The integrity of physical barrier is fundamental to the animal health and productivity since it prevents the translocation of toxins and pathogens from the rumen into the blood; (3) the immune barrier, which is composed of the intestinal-associated immune cells and their secretion of cytokines. Under the physiological conditions, the immune cells, especially the innate immune cells, play the key roles in the maintenance of intestinal homeostasis by preventing inappropriate adaptive immune responses. For example, the activation of the Toll-like receptor (TLR) 10 signaling enhanced the tolerance of rumen epithelium to the rumen fluid microbiota by suppressing the expressions of pro-inflammation cytokines (Shen et al., 2016). The activation of the TLR2 signaling in intestinal epithelium enhanced its barrier function via promoting the expression of zonula occludens-1 (ZO-1) (Cario et al., 2004). So far, many factors, ranging from the environmental factors of the microbial ecological niche to the physiological conditions of the host, have been reported to impact the functions of these barriers. Among them, SCFAs (mostly butyrate, acetate, and propionate) have received the greatest attentions. In the rumen, SCFAs have been reported to influence the function of the physical barrier, such as the integrity of rumen epithelium, renewal of epithelial cells and the expression of tight junction proteins (Gui and Shen, 2016; Greco et al., 2018). In the colon, SCFAs have been reported to promote the proliferation and renewal of epithelial cells (Guilloteau et al., 2010); to influence the size, and function of regulatory T cells (Tregs) via binding to the G-protein-coupled receptors (GPRs) and histone deacetylase (HDACs) expressed in the epithelium (Puertollano et al., 2014); to regulate the epithelium motility and permeability via the hormono–neuroimmune system (Perry et al., 2016). In addition, in the human intestine, SCFAs have been reported to suppress the expression of virulence genes in the opportunistic pathogens (Ohland and Jobin, 2015). However, the various parts of rumen barriers do not work separately. On the contrary, changes in environmental factors have global effects on the function of these barriers. For example, any excess amount of SCFAs generated in the rumen leads to reconstruct the epimural microbiota and the microbial barrier, damage the epithelial structure and the physical barrier, and concomitantly, express inflammatory cytokines in the rumen epithelium (Liu et al., 2013; Wetzels et al., 2015) and impair the immune barrier. Hence, the study of the global effects of ruminal SCFAs on the function of rumen barriers is valuable in order to obtain a comprehensive view of the interactions between ruminal SCFAs and rumen barriers and, furthermore, will provide potential regulation methods for animal health and growth.

The ratio of dietary concentrate is well known to influence the concentrations of ruminal SCFAs. In the current study, we have collected the ruminal epithelium and epimural microbiota from the rumen of goats receiving diet with three ratios of concentrate diet, i.e., 10% (LC), 35% (MC), and 65% (HC). Previous studies showed that MC diet promotes the performance in structural integrity, nutrient absorption and cell refreshing of rumen epithelium better than that of the LC and HC group (Yan et al., 2014; Gui and Shen, 2016), and that both of LC and HC diets had negative effects on the integrity of the rumen epithelium, indicating the strongest barrier function of MC group among three groups (Hu et al., 2018). Accordingly, in order to know the effects of the increase of ruminal SCFAs on the barrier function of rumen epithelium in the present study, we compared the barrier functions between the LC and MC group by using LC group as the control, and compared the barrier functions between the MC and HC group by using MC group as the control. Via the simultaneous measurement of the responses from three parts of the rumen barrier, we hope to gain better insights into the effects of ruminal SCFAs on the function of rumen barriers.

## Materials and Methods

### Ethics Statement

This study was carried out in accordance with the recommendation of the Regulations for the Administration of Affairs Concerning Experimental Animals (No. 588 Document of the State Council of P.R. China, 2011). This protocol was approved by Nanjing Agricultural University.

### Experiment Design

Fifteen male goats (Boer × Yangtze River Delta White, aged 4 months) were randomly allocated into three groups and received a diet of 35% hay plus 65% concentrate (HC group, *n* = 5), a diet of 65% hay plus 35% concentrate (MC group, *n* = 5), and a diet of 90% hay plus 10% concentrate (LC group, *n* = 5) (Table 1). All goats were fed with two equal portions of the designated diet at 0800 and 1700 daily for 28 days. Water was freely available to all goats during the experimental period. On day 29, the goats were killed at a local slaughterhouse.

**TABLE 1:** Dietary compositions used in this study.

**Item**	**HC^a^**	**MC^a^**	**LC^a^**
			
	**Guinea grass + corn**	**Guinea grass + corn**	**Guinea grass**
**Dietary intake**			
Total DMI, g/d	758.73	773.61	783.3
NDF, g/d	302.16	414.66	527.07
NFC^b^, g/d	375.44	218.71	110.31
Initial body weight, kg	16.2 ± 2.3	16.3 ± 1.6	16.3 ± 2.0
**Ingredient, % Of Dm**			
Guinea grass	35	65	90
Corn	55	25	0
Soya bean meal	8	8	8
Additive^c^	2	2	2
**Chemical composition**			
DM,%	87.78	89.46	90.63
Crude protein, %DM	10.52	9.98	9.61
Crude fat, %DM	2.86	2.94	3
Crude fiber, %DM	12.89	22.84	29.51
Crude ash, %DM	3.98	5.2	6.02
NFC, %DM	49.48	28.27	14.08
NDF, %DM	33.16	53.6	67.29
ME^d^, Mcal/kg of DM	2.42	2.72	3.35

*Concentrate = corn + soya bean meal. DMI, dry matter intake; ME, metabolizable energy; NDF, neutral detergent fiber; NFC, non-fibrous carbohydrate; DM, dry matter. ^*a*^The values are means ± SE. ^*b*^NFC = 100 – (NDF + CP + crude fat + ash). ^*c*^The additive was composed of calcium phosphate, limestone, trace mineral salt, and vitamin premix (vitamins A, D, and E). ^*d*^ME = total digestible nutrient × 0.04409 × 1.01–0.45 (NRC, 2000).*

### Sample Collection and Determination of SCFA Concentrations, pH and Osmolarity in the Rumen

On day 29, all goats were slaughtered at 6 h after receiving the morning feed. Ruminal content (30 mL) were strained through a 4-layer cheesecloth and immediately subjected to pH measurement by using a pH meter (Mettler-Toledo Delta 320, Halstead, United Kingdom). A 5% HgCl_2_ solution was added to the fluid samples, which were subsequently stored at −20°C for the determination of SCFA concentration and osmolarity. Rumen tissue from the ventral blind sac was quickly excised and washed in ice-cold phosphate-buffered saline (PBS; pH 7.4). The epithelium was subsequently separated from the muscle layers and cut into 1–2 cm^2^ pieces. For each animal, five pieces of rumen epithelium were immediately fixed in 4% paraformaldehyde (PFA) (Sigma, St. Louis, MO, 123 United States) for morphometric analyses. Ten pieces were stored at −20°C for the later extraction of microbial DNA. Ten pieces were stored at −80°C for the later extraction of epithelial RNA.

Ruminal SCFAs concentrations were measured by using a gas chromatograph (HP6890N, Agilent Technologies, Wilmington, DE) as described by Yang et al. (2012). 10 mL of rumen fluid was centrifuged at 18,000 g for 20 min at 4°C (Eppendorf Centrifuge 5424 R, Eppendorf AG, Hamburg, Germany). The supernatant was collected and the osmolarity was measured by using an osmometer (Osmomat 030-D, GONOTEC Berlin, Germany).

### Morphological Analysis of Rumen Epithelium

The density, length and width of ruminal papillae were measured according to the description of Malhi et al. (2013). In brief, 1 cm^2^ of PFA fixed ruminal epithelium was used to count for the papillae density (number/cm^2^). Fifteen papillae in each of the PFA fixed epithelial sample were used to measure papillae length and width by using a sliding caliper.

### Microbial DNA Extraction and 16S rRNA Gene Sequencing

To detach the tightly attached microbes, twenty pieces of ruminal epithelium were placed in a 15 ml tube with 7 ml PBS and several plastic beads and moderately shaken on a vortex for 30 s. The ruminal epithelium was moved to a new tube and processed with the detaching step for two more times. Subsequently, the metagenomic DNA was extracted from the PBS mixture by using a Bacterial DNA Kit (Omega, Shanghai, China). The DNA concentration was determined in a NanoDrop 1000 (Thermo Fisher Scientific, Wilmington, DE, United States) and stored at −20°C until further processing.

The amplicon library was prepared by polymerase chain reaction (PCR) amplification of the V4 region of the 16S *rRNA* gene. The universal primers 515F and 806R, including TruSeq adapter sequences and indices, were employed in the PCRs. All libraries were sequenced by using an Illumina HiSeq2500 platform (Illumina, San Diego, CA, United States) at Biomarker Technologies, Beijing, China.

### Composition Analysis of Epimural Microbes by Using 16S rRNA Gene Sequencing Data

Paired reads were filtered for quality (Q30) and joined by using FLASH version 1.2.11 (Magoc and Salzberg, 2011). Sequences that contained read lengths shorter than 250 bp were removed by means of PRINSEQ v0.20.4 (Schmieder and Edwards, 2011). The remaining sequences were then classified into operational taxonomic units (OTUs) by using QIIME 1.9.0 (Caporaso et al., 2010) at a 97% similarity threshold. OTUs whose counts were more than 3 in at least one of the samples were hierarchically summed at all taxonomic levels, and the counts were normalized to the relative abundance for each sample. The diversity of the microbial communities was estimated by using the R program phyloseq package (McMurdie and Holmes, 2013).

### Covariation of SCFA Concentration and Microbial Abundance

The relationships between the abundance of OTUs and the concentrations of SCFAs were explored by canonical correspondence analysis (CCA) in the R program vegan package (Oksanen et al., 2016). Subsequently, the R program ggplot2 package (Wickham, 2009) was used to generate the visual interpretation (biplot) of the gene-microbe relationships.

### Metagenome Shotgun Sequencing and Function Comparison of Epimural Microbes

The integrity of microbial DNA was evaluated on 1.0% agarose gel. Metagenomic DNA libraries were constructed by using the TruSeq DNA Sample Prep kit (Illumina, San Diego, CA, United States). Libraries were sequenced via paired-end chemistry (PE150) on an Illumina Higseq X Ten platform (Illumina, San Diego, CA, United States) at Biomarker Technologies, Beijing, China.

Raw reads were first filtered by using FastX v0.0.13 (Gordon and Hannon, 2010), with a quality cutoff of 20, and reads shorter than 30 bp being discarded. The high-quality reads that were likely to originate from the host were removed by using DeconSeq v0.4.3 (Schmieder and Edwards, 2011), with the NCBI goat genome sequence as the reference. The remaining reads were subsequently assembled into scaftigs by using IDBA-UD (Peng et al., 2012) with the standard parameters. Genes were predicted from the scaftigs by means of FragGeneScan (Rho et al., 2010). The predicted genes of each sample were then annotated to Kyoto Encyclopedia of Genes and Genomes (KEGG) ontology (KO) databases via the KEGG Automatic Annotation Server (KAAS) (Moriya et al., 2007).

Predicted genes from all samples were gathered together to form a large gene set. BLAT v35 was used to construct the non-redundant gene set. Any two genes with more than 95% identity and more than 90% coverage of the shorter gene were picked out, and subsequently, the shorter one was removed from the large gene set. High-quality reads of each sample were mapped to the non-redundant gene set by using Bowtie2 v2.3.4 (Langmead and Salzberg, 2012) with default parameters. MarkDuplicates in the Picard toolkits version 2.0.1 was used to remove the duplicates in the reads, and then genomeCoverageBed in BEDTools 2.26.0 was used to calculate the gene coverage. The RPKM of the gene, calculated by [gene coverage × 10^6^/(total mapped reads × gene length)], was used to normalize the gene abundance between the treatments. The abundances of KOs were received by summing up the abundance of genes with the same KO number, and then, compared by using R program DeSeq2 package (Love et al., 2014). Since the multiple comparisons (LC vs. MC and MC vs. HC) were proceed in this study, the Bonferroni-Holm (BH) correction has been done for the *p.adjust* values received from DeSeq2 comparison (the corrected values were termed as *p.correct* in this study). Differences were considered significant when *p.correct* < 0.05 and the differences of KO abundance were more than two times between the groups.

In this study, the abundance of the KEGG pathway was transformed from the detected kinds of differentially abundant genes, which were located in the corresponding KEGG pathway, within the sample. Subsequently, the abundance of the microbial KEGG pathway was compared and visualized by using R program heatmap3 v1.1.4 (Zhao et al., 2014) with the complete clustering method.

### Epithelial RNA Extraction and Transcriptome Sequencing

Total RNA was extracted from the ruminal epithelium by using the RNAeasy Mini Kit (Qiagen, Shanghai, China). RNA was quantified by using the NanoDrop 1000 spectrophotometer, and its integrity was evaluated by means of the RNA 6000 Assay Kit of the Agilent Bioanalyzer 2100 system (Agilent Technologies, CA, United States). High-quality RNA (RNA integrity number > 9.0) was processed by applying the NEB Next Ultra RNA Library Prep Kit (New England Biolabs, Beijing, China). All libraries were sequenced via paired-end chemistry (PE125) on an Illumina HiSeq2500 platform (Illumina, San Diego, CA, United States) at Biomarker Technologies, Beijing, China.

### Transcriptome Assembling, Differentially Expressed Gene Identification, and KEGG Enrichment Analysis

Low-quality reads were first removed by using PRINSEQ v0.20.4. The NCBI goat genome annotation release version 101 was applied to construct the reference genome by means of Bowtie v1.2.0 (Langmead et al., 2009). High-quality reads were mapped to the reference genome by using TopHat v2.1.0 (Kim et al., 2013) with standard parameters. for each sample, the gene expression level was estimated and normalized to the reads per kilobase of exon model per million mapped reads (RPKM) by means of Cufflinks v2.2.1 (Trapnell et al., 2010). In this study, only genes with more than 1 RPKM in at least one group of the samples were considered to be expressed. Similar with the metagenomic analysis, the DeSeq2 package was utilized to detect the differentially expressed genes between the groups. After the BH correction, the differences were considered significant when *p.correct* < 0.05 and the differences of genes expression were more than two times between the groups. The R program clusterProfiler package (Yu et al., 2012) was utilized to perform the KEGG enrichment analysis for the differentially expressed genes between the groups. The sets of upregulated genes and downregulated genes were used in the enrichment analysis, separately. Finally, the enrichment results were visualized by using the R program ggplot2 package.

### SCFAs Regulatory Network Construction

The highly correlated genes of SCFAs were identified by computing the Spearman correlation coefficient (SCC) between SCFA concentration and gene expression across the groups in the R program. Only expressed genes were included in the correlation analysis. A threshold for the SCC value larger than 0.8 and a *p*-value less than 0.01 was employed to identify significantly related genes. Accordingly, two SCFAs regulatory networks were constructed based on the SCCs of genes and SCFAs between the LC group and MC group (referred to as the MC-LC SCFA regulatory network) and the SCCs of those between the MC group and HC group (referred to as the HC-MC SCFA regulatory network). Subsequently, the genes that were highly correlated to the SCFAs were picked and annotated against the KEGG databases. Finally, the correlation networks were visualized by means of cytoscape 3.4.0 (Shannon et al., 2003).

### Reverse Transcription Quantitative PCR (RT-QPCR) Verification of Target Genes

An aliquot of 2000 μg RNA, random hexamer primers (Invitrogen, Shanghai, China) and moloney murine leukemia virus (M-MLV) reverse transcriptase (Fermentas, Burlington, ON, Canada) were employed to synthesize the cDNA. RT-QPCR was performed by using the StepOne Plus real-time PCR system (Applied Biosystems, Den Ijssel, Netherlands) and SYBR-Green (Roche, Shanghai, China) for detection. The glyceraldehyde-3-phosphate dehydrogenase (GADPH) was chosen as the stably expressed reference gene. The primers of these genes were designed in this study by using Primer 5 and the available mRNA sequences in NCBI (Supplementary Table S1). Amplification efficiencies of the primers were determined by means of a dilution series of epithelial cDNA. All samples were run in triplicate, and the data were analyzed according to the 2^–ΔΔCT^ method (Livak and Schmittgen, 2001). The identity and purity of the amplified products were checked by analysis of the melting curves obtained at the end of the amplification. Linear regression analysis was applied to identify the relationships between the RT-QPCR results and RNA-seq results.

### Statistical Analysis of SCFAs Concentration, pH, Osmolarity, Morphological Traits, and RT-QPCR Results

The BH corrected two-tailed *t*-test was used to analyze the difference of SCFAs concentration, pH, osmolarity, morphological trait and RT-QPCR results between the groups (Tables 2, 3). The differences were considered significant when *p.correct* < 0.05. The Pearson correlation coefficient (PCC) was calculated to detect the relevance between the total SCFA concentration and ruminal osmolarity. Relevance was considered significant when PCC > 0.8 and *p.correct* < 0.05.

**TABLE 2:** Effects of LC, MC, and HC diets on the ruminal papillae characteristics.

**Item**	**LC^a^**	**MC^a^**	**HC^a^**	***p.correct* (LC vs. MC)^b^**	***p.correct* (MC vs. HC)^b^**
Length (mm)	1.37 ± 0.03	3.59 ± 0.11	2.53 ± 0.08	<0.01	<0.01
Width (mm)	1.42 ± 0.05	1.34 ± 0.01	1.24 ± 0.08	0.16	0.50
Density (number/cm^2^)	79.67 ± 1.69	108.33 ± 5.66	88.67 ± 6.04	<0.01	0.09

*^*a*^Value is mean ± standard error (SE). ^*b*^Bonferroni-Holm corrected two-tailed t-test.*

**TABLE 3:** Effects of LC, MC and HC diets on the concentrations of SCFAs, pH, and osmolarity in the goat rumen.

**Item**	**LC^a^**	**MC^a^**	**HC^a^**	***p.correct* (LC vs. MC)^b^**	***p.correct* (MC vs. HC)^b^**
Acetate (mM)	70.62 ± 1.91	77.41 ± 2.23	84.15 ± 2.76	0.05	0.19
Propionate (mM)	14.80 ± 0.31	17.31 ± 0.62	19.91 ± 0.60	0.01	0.03
Butyrate (mM)	4.92 ± 0.15	7.24 ± 0.24	9.89 ± 0.17	<0.01	<0.01
Total SCFA (mM)	90.34 ± 2.22	101.96 ± 2.11	113.95 ± 2.27	0.01	0.01
pH	6.66 ± 0.02	6.35 ± 0.01	6.11 ± 0.02	<0.01	<0.01
Osmolarity (mosm/L)	278.33 ± 2.20	300.00 ± 2.33	353.00 ± 2.69	<0.01	<0.01

*^*a*^Values are mean ± standard error (SE). ^*b*^Bonferroni-Holm corrected two-tailed t-test.*

## Results

### Morphological Analysis of the Rumen Epithelium

Compared with the LC group, the length and density of the ruminal papillae were increased in the MC group. On the contrary, the length was decreased in the HC group, compared with the MC group (Table 2).

### Comparisons of Ruminal SCFA Concentrations, pH, and Osmolarity Across the Groups

Compared with the LC group, the concentrations of acetate, propionate, butyrate and total SCFA were increased by 9, 16, 47, and 13% in the MC group, respectively (Table 3). Meantime, the concentrations of propionate, butyrate and total SCFA showed the significant changes between the LC and MC groups. Compared with the MC group, the concentrations of acetate, propionate, butyrate and total SCFA were increased by 8, 15, 37, and 12% in the HC group, respectively. Meantime, the concentrations of propionate, butyrate and total SCFA showed the significant changes between the MC and HC groups. The ruminal pH was consistently decreased with increases of dietary concentrate. The ruminal pH was significantly reduced, whereas the osmolarity was significantly raised with the increase of dietary concentrate. According to the Pearson correlation analysis, the ruminal osmolarity was significantly correlated with the total SCFA concentration (PCC = 0.865, *p* = 0.01).

### Composition and Diversity of Epimural Microbes

**FIGURE 1:**
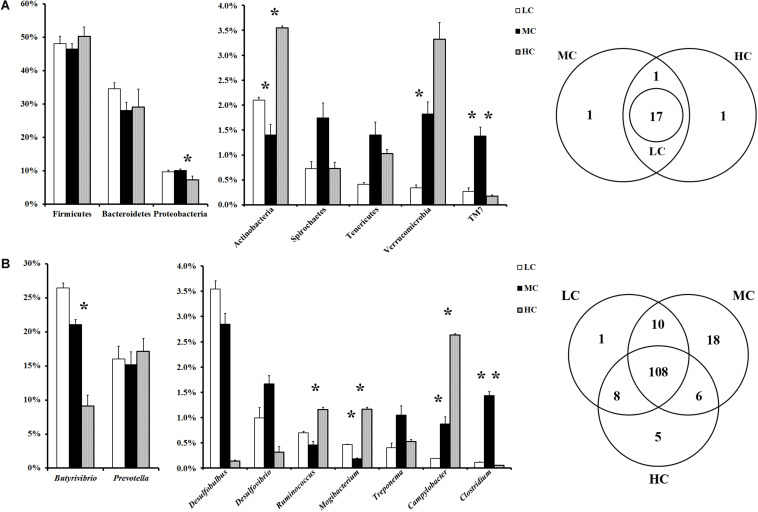
**(A)** Phylum-level comparison of bacterial OTUs across groups; **(B)** genus-level comparison of bacterial OTUs across the groups. MC group was used as the control for the comparison; “^∗^” located between first two columns indicates a *p* < 0.05 in the *t*-test of MC and LC groups; “^∗^” located between next two columns indicates a *p* < 0.05 in the *t*-test of MC and HC groups.

At the phylum level, a total of 20 prokaryotic phyla were identified at a 97% similarity, and 17 of them were common to all groups (Figure 1A). Firmicutes (46.5–50.2%), Bacteroidetes (28.1–34.5.0%), and Proteobacteria (7.3–10.1%) were most abundant among all microbial communities. Compared with the LC group, Verrucobacteria showed the most significant increase (increased by 4.3 times), whereas Actinobacteria showed the most significant reduction (decreased by 33%), in the MC group. Compared with the MC group, Actinobacteria showed the most significant increase (increased by 1.5 times), whereas Proteobacteria showed the most significant reduction (decreased by 27%), in the HC group. At the genus level, in total, 156 genera were detected in the sequences. Among them, 108 genera were common to all groups (Figure 1B). The abundances of all genera in the groups are shown in Supplementary Table S2. *Butyrivibrio* was the most abundant genus in both the MC and LC groups, whereas *Prevotella* was the most abundant genus in the HC group. Compared with the LC group, *Ruminobacter* and *Anaerostipes* showed the most significant increase (increased by 74 times and 46 times, respectively), whereas *Sphingobium*, *Streptococcus*, and *Acinetobacter* were the most significantly reduced (decreased by 99, 99, and 98%, respectively), in the MC group. Compared with the MC group, *Sphingobium*, *Acinetobacter*, and *Streptococcus* exhibited the most significant increase (increased by 135 times, 112 times, and 44 times, respectively), whereas *Bifidobacterium*, *Microbacterium*, *Anaerostipes* and *Clostridium* were the most significantly reduced (decreased by 99, 99, 98, and 96%, respectively), in the HC group.

Associated with the increase in the concentration of total SCFA, the diversity of the epimural microbiota presented the curve with a gradual increase from the LC group to the MC group, and a sharp decline from the MC group to the HC group (Supplementary Figure S1).

### Relationships Between SCFA Concentrations and Relative Abundance of Microbe Genera

**FIGURE 2:**
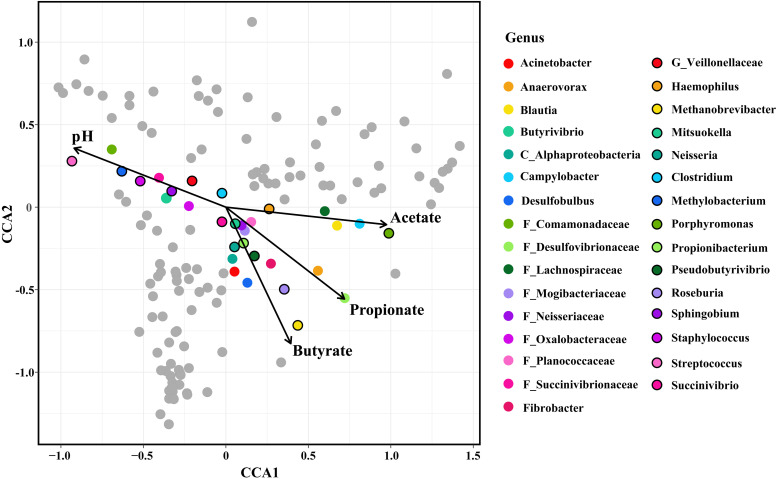
Constrained correspondence analysis (CCA) revealing the correlations between the abundance of the microbial genera and the concentrations of ruminal SCFAs/the value of ruminal pH. The genera, whose relative abundance was highly related to the SCFAs concentration/ruminal pH in the CCA, were colored. The length of the arrow indicated the importance of SCFAs/pH in the shaping of the epimural microbiota, and the vertical distance of the special genera to the arrow indicated the correlation between SCFAs concentrations/pH and the relative abundance of microbial genera.

CCA showed that the relative abundances of OTUs belonging to 25 genera were highly related to the concentrations of ruminal SCFAs. Moreover, the relative abundances of OTUs belonging to 10 genera were highly related to the ruminal pH (Figure 2).

### Functional Comparisons of Epithelium Microbes Across the Groups

**FIGURE 3:**
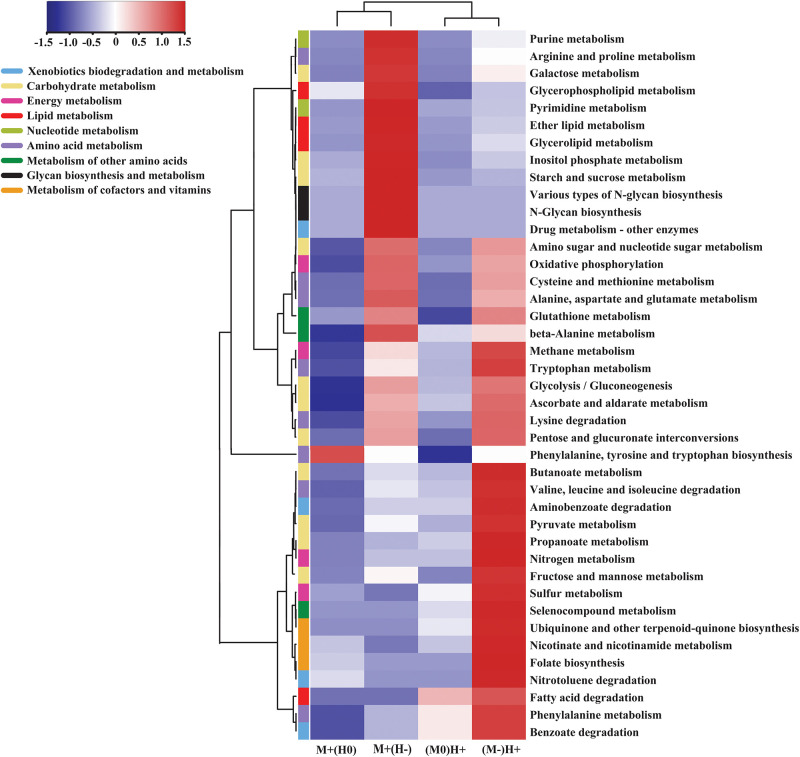
Heatmap comparing the kinds of gene, which has the significantly different abundance across the groups, on the specific KEGG metabolism pathway. M + (H0) indicates that, in this column, the abundance of genes was significantly increased in the MC group compared to the LC group, and meantime, had no significant changes between MC group and HC group; M + (H-) indicates that, in this column, the abundance of genes was significantly increased in the MC group compared to the LC group, and meantime, was significantly decreased in the HC group compared to the MC group; M0(H+) indicates that, in this column, the abundance of gene had no significant changes between LC group and MC group, and meantime, was significantly increased in the HC group compared to the MC group; M-(H +) indicates that, in this column, the abundance of genes was significantly decreased in the MC group compared to the LC group, and meantime, was significantly increased in the HC group compared to the MC group.

Metagenome shotgun sequencing generated a total of 156 G high-quality data. Among them, approximately 20 G data, which were likely to originate from the host, were excluded from the datasets. On average, 74% of the clean data was successfully assembled into the scaftigs. Subsequently, an average of 176,546, 206,490, and 211,140 open reading frames (ORFs) were detected within the LC, MC, and HC groups, which totally annotated to 6,605 KOs (Supplementary Table S3). Compared with the LC group, the relative abundance of 1,415 KOs was significantly upregulated, and the relative abundance of 534 KOs was significantly downregulated, in the MC group. Compared with the MC group, the relative abundance of 600 genes was significantly upregulated, and the relative abundance of 847 genes was significantly downregulated, in the HC group. Finally, these differentially abundant genes were annotated to 41 KEGG pathways of metabolism (Figure 3).

In the comparisons of the abundance of the metabolism pathways, we observed that galactose metabolism, starch and sucrose metabolism, three kinds of lipid metabolism, nucleotide metabolism and glycan biosynthesis and metabolism were upregulated in the MC group compared with the LC group, whereas both of them were downregulated in the HC group compared with the MC group. On the contrary, glycolysis, pyruvate metabolism, propanoate metabolism, butanoate metabolism and the metabolism of cofactors and vitamins were downregulated in the MC group compared with the LC group, whereas all of them were upregulated in the HC group compared with the MC group (Figure 3).

### Enriched KEGG Pathways of Differentially Expressed Genes Related to the Immune System and Cellular Processes in the Rumen Epithelium

Transcriptome sequencing generated a total of 129 G raw data. On average, 83% of the high-quality reads were successfully mapped to the NCBI goat genome. Finally, in total, 11,131 genes were detected as being expressed in the rumen epithelium of these goats (Supplementary Table S4). Compared with the LC group, 374 genes were significantly upregulated, and 478 genes were significantly downregulated, in the MC group. Compared with the MC group, 267 genes were significantly upregulated, and 283 genes were significantly downregulated, in the HC group.

**FIGURE 4:**
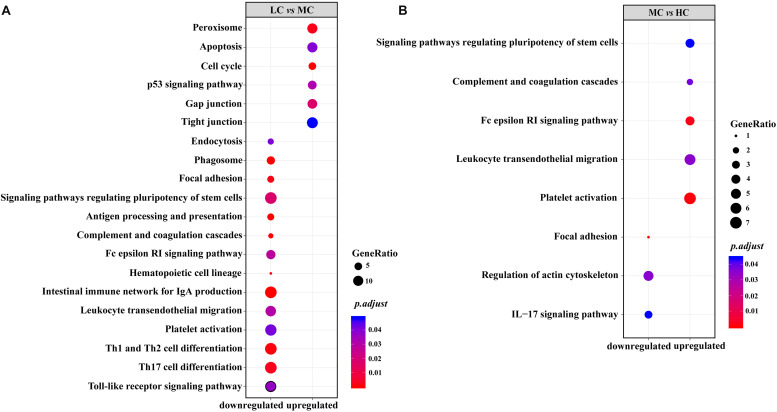
**(A)** Upregulated/downregulated KEGG pathways related to the immune system and cellular process in the rumen epithelium of the MC group, compared with those in the LC group; **(B)** upregulated/downregulated KEGG pathways related to the immune system and cellular process in the rumen epithelium of the HC group, compared with those in the MC group.

In the KEGG enrichment analysis, we observed that, in the MC group, all enriched immune-related pathways were downregulated in comparison with those in the LC group (Figure 4). The downregulated genes located on the enriched pathways, when the diets shifted from LC to MC, were listed in Supplementary Table S5. On the contrary, in the HC group, excepting for the IL-17 signaling pathway, all enriched immune-related pathways were upregulated in comparison with those in the MC group. The upregulated genes located on the enriched pathways, when the diets shifted from MC to HC, were listed in Supplementary Table S6. In the part of cellular process, in the MC group, all enriched pathways related to the cell growth and death (apoptosis, cell cycle and p53 signaling pathway), gap junction, tight junction and peroxisome were upregulated, while endocytosis, phagosome, focal adhesion and signaling pathways regulating pluripotency of stem cells were downregulated, in comparison with those in the LC group (Figure 4A and Supplementary Table S5). However, only two pathways related to the cellular community (signaling pathways regulating pluripotency of stem cells and focal adhesion) and one pathway related to the cell motility (regulation of actin cytoskeleton) were enriched in the HC group, in comparison with those in the MC group (Figure 4B and Supplementary Table S6).

### Comparisons of MC-LC and HC-MC SCFA Regulation Networks

**FIGURE 5:**
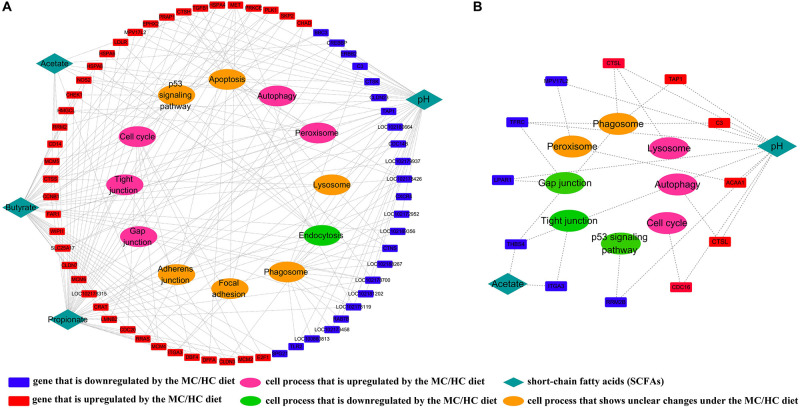
**(A)** Regulatory effects of ruminal SCFAs and ruminal pH on cellular processes by stimulation of the expression of corresponding genes in the MC group, compared with those in the LC group; **(B)** regulatory effects of ruminal SCFAs and ruminal pH on cellular processes by stimulation of the expression of corresponding genes in the HC group, compared with those in the MC group.

According to the SCCs between the MC and LC groups, the concentrations of ruminal acetate, butyrate, and propionate, together with the ruminal pH, had a high possibility of affecting the expression of genes located on 12 pathways of the cellular process. Moreover, the results suggested that genes associated with the cell cycle, tight junction, gap junction, peroxisome, and autophagy were promoted, whereas those associated with endocytosis were suppressed by the increased SCFA concentrations and the decreased pH of the MC group, compared with the LC group (Figure 5A). However, according to the SCC between the HC and MC groups, the further increase in the acetate concentration and the further decrease in the ruminal pH had a high possibility of suppressing the cellular functions concerning the gap junction, tight junction, and p53 signaling pathway, whereas they promoted the cellular functions concerning the lysosome, autophagy and cell cycle, through their impacts on the expressions of the corresponding genes (Figure 5B).

### Confirmation of High-Throughput Sequencing Results by RT-QPCR

To validate high-throughput sequencing results, we compared the expression of 11 rumen epithelial genes which showed the significantly changed both between LC and MC groups and between MC and HC groups (Supplementary Tables S5, S6) by using RT-QPCR method, and the results are shown in Supplementary Table S7. In general, the expression analyses of these genes verified the significant difference discovered by RNA-seq. Linear regression analysis showed that the RT-QPCR results were highly consistent with the RNA-seq results (*R*^2^ = 0.80) (Supplementary Figure S2).

## Discussion

Previous studies of humans have shown that an increase in the ratio of Firmicutes to Bacteroidetes is associated with the ability to extract more energy from food (Jumpertz et al., 2011). If it is also applied to the rumen epimural microbiota, the acid environment induced the decrease on energy-extraction ability of the epimural microbiota might be part of the reason for the sharp decline of the microbial diversity in the HC group, in comparison with that of MC group. Next, the 16S *rRNA* sequencing data, metagenomic data and CCA results all indicated the importance of genera *Sphingobium, Acinetobacter*, and *Streptococcus* in the SCFA-microbe-host axis. So far, most of the known members in the genera *Acinetobacter* and *Streptococcus* exhibit both a pathogenic lifestyle and a commensal lifestyle in the GI tract. The study has shown that genomic plasticity enabling quick adaptation to environmental stress is a necessity for their pathogenic lifestyle, whereas the stability is a necessity for their commensal lifestyle (Kilian et al., 2014). Accordingly, we infer that, in an acid environment, the intermediate metabolites of the ruminal microbiota, such as lactate, promotes the growth of opportunistic pathogens, such as *Streptococcus*, in the rumen epithelium. In order to enhance the genomic plasticity of stress resistance, these opportunistic pathogens recruit microbes to form a multi-species biofilm and, subsequently, increase the ability of stress resistance in the biofilm by using the quorum-sensing system (Nadell et al., 2009; Belkaid and Hand, 2014). Under these conditions, the opportunistic pathogens are prone to the pathogenic lifestyle and, thereby, promote immune responses in the rumen epithelium. On the contrary, in the MC group, the moderate increases of ruminal SCFAs promote the growth of the stratum corneum, whose keratin is an important nutrient for epimural microbes (Cheng et al., 1979) and, thereby, promote the diversification of epimural microbiota and its gene pool. According to ecological theory, the more diverse a community is, the more stable the ecosystem is. Thus, the moderate concentration of ruminal SCFAs promote the stability of the micro-ecosystem on the surface of the rumen epithelium and, thereby, promote the commensal lifestyle of the opportunistic pathogens in the rumen epithelium. Moreover, the increases of the ruminal butyrate may also help the host to suppress the expression of the virulence genes in the opportunistic pathogens (Ohland and Jobin, 2015). In short, our results indicate that, rather than having impacts on the colonization of opportunistic pathogens in the rumen epithelium, ruminal SCFAs affect the diversity of epimural microbiota and the lifestyle of opportunistic pathogens. However, further studies of meta-transcriptome and meta-metabolome are needed to reach comprehensive views concerning the interactions between ruminal SCFAs and epimural microbes.

According to the changes in the microbial metabolism pathways (Figure 3), our study primarily indicated that the epimural microbiome had different metabolism network responses to the dietary shifts from LC to MC, compared with the responses to the dietary shifts from MC to HC. This result is consistent with the results on the structure of microbial community. So far, there have been many reports on the kinds of microbial metabolites that modulate the barrier function of intestinal epithelium. Among them, SCFAs are the most studied metabolites, which promoted the barrier functions by promoting the production of protective mucus and IgA, regulating the differentiation of Tregs, and suppressing the production of inflammatory mediator nuclear factor κB (NF-κB) (Ohland and Jobin, 2015). In addition, fose, a metabolite of *Bacteroides thetaiotaomicron*, inhibited the expression of the virulence factor *ler* in pathogenic *E. coli* within the colon (Pacheco et al., 2012). Trp metabolites provided the colonization resistance to the pathogenic fungus *Candida albicans* in the mice gut (Zelante et al., 2013). Lipopolysaccharides (LPS), the metabolites of gram negative bacteria, impaired the integrity of rumen epithelium in the cow with subacute ruminal acidosis (SARA) (Kleen et al., 2003; Emmanuel et al., 2007). Our study showed, when the diets shifted from the LC to the MC, the newly appeared genes and the significantly increased genes were the pathway enzymes in (1) the metabolism of nucleotide and glycan. The nucleotide and glycan are the important components of microbes. Therefore, the increases of these metabolites could provide the materials for the growth of microbes; (2) the metabolism of lipids into fatty acids, and the metabolism of starch, sucrose and galactose into glucose. The fatty acids and glucose are the energy substrates of microbes. Therefore, the increases of these metabolites could provide the energy for the growth of microbes. When the diets shifted from the MC to the HC, the newly appeared genes and the significantly increased genes were the pathway enzymes in the fermentation of glucose and fatty acids into SCFAs and some other products, such as lactate and ethanol. The excessive increases of fermentation products impair the integrity of rumen epithelium and cause the SARA. It should be highlighted that our results, obtained from the statistical analysis, can only give the hints based on the statistical analysis. The real effects of the microbial metabolism on the function of rumen barrier, as well as the mechanisms, need further studies.

In the rumen epithelium, the results of our transcriptomic data showed that, associated with the increases on the ruminal SCFAs, the responses of immune components (including innate and adaptive immune cells, platelet, hematopoietic cell lineage and leukocyte) were decreased from the LC group to the MC group, whereas all of them were increased from the MC group to the HC group. Notably, the diversity of the gene pools related to the disease infection of the epimural microbes showed the opposite trend to the degree of immune responses in the rumen epithelium. Since most of the microbial pattern recognition receptors (MPRRs), which receive and deliver the signals from the microbes to the immune cells, are located in the basal of the rumen epithelium (Malmuthuge et al., 2012), and since, concomitantly, the changes in the thickness of the rumen epithelium are opposite to the responses of immune cells in the rumen epithelium, as revealed by the morphological measurement, we infer that the structure of the rumen epithelium and the activities of the epithelial cells play important roles in the shaping of the immune responses in the rumen epithelium through their effects on the reduction of dangerous signals from epimural microbes to the rumen epithelium. However, further evidence to support this hypothesis is required.

To date, the structure and function of the rumen epithelium is well known as being affected by the concentrations of ruminal SCFAs, the ruminal osmolarity and the ruminal pH. For example, butyrate promotes the thickening of the rumen epithelium by stimulating its growth and renewal (Malhi et al., 2013). *In vitro* increase of SCFA concentration at pH 6.1 upregulated the mRNA expression of tight junctions Cldn-1 and 4 (Greco et al., 2018). Ruminal osmolarity, mainly determined by the concentrations of ruminal SCFAs, influenced the paracellular resistance, apical Na^+^-H^+^ exchange activity and integrity of rumen epithelium (Bennink et al., 1978; Schweigel et al., 2005; Lodemann and Martens, 2006). Increase of SCFAs concentration and decrease of ruminal pH enhanced the expressions of genes related to the cell proliferation and apoptosis in the rumen epithelium (Sun et al., 2018). In our study, in order to check the effects of ruminal SCFAs on epithelium structure, we constructed the SCFA regulatory network by using transcriptomic data. We observed that the tight junction, gap junction, and p53 signaling pathway were promoted by the increased concentrations of ruminal SCFAs and the decrease of ruminal pH from the LC group to the MC group, whereas all of them were suppressed by the further increase in the concentration of acetate and the decrease of ruminal pH from the MC group to the HC group. The upregulation of the tight junction and gap junction effectively limits the invasion of dangerous signals to the animal tissue, thereby contributing to the reduction of inflammatory responses in the rumen epithelium. The upregulation of the p53 signaling pathway, together with the upregulation of cell proliferation, promotes the cell refreshing in the MC group. These data are in agreement with the increased rumen papillae length and papillae density in MC group of current study, and consistent with the data in our previous study (Gui and Shen, 2016), which reported the moderate upregulation of ruminal SCFAs concentration induced the increase of cell apoptosis and p53 gene expression in rumen epithelium of goats. Taken together, these studies indicate the epithelial homeostasis and molecular adaptation of rumen epithelium to adequate concentrate intake is associated to moderate concentration of SCFA and pH in rumen. But, excess amount of SCFA and acidic pH, induced by high concentrate intake, may cause the damage of tight junction and gap junction in rumen epithelium and impair epithelial homeostasis (Greco et al., 2018).

Our examination of the regulatory networks additionally demonstrated that endocytosis was suppressed by the increased concentrations of ruminal SCFAs from the LC group, compared to the MC group, whereas it was not significantly changed with a further increase in the concentration of ruminal acetate and a further decrease in the ruminal pH, from the MC group to the HC group. Endocytosis is a mechanism for cells to remove ligands, including MPRRs, from the cell surface. Therefore, the upregulation of endocytosis contributes to the suppression of the immune response to non-pathogenic microbes in the rumen epithelium. Moreover, peroxisomes are exclusively promoted by increased SCFA concentrations and a decreased pH, from the LC group to the MC group. The upregulation of peroxisomes, which play an important role in lipid homeostasis and free radical detoxification, is essential for the maintenance of epithelial integrity. Whereas lysosomes are exclusively promoted by an increased acetate concentration and a decreased pH in the MC group compared to the HC group. The upregulation of lysosomes, which help the cells to kill and degrade invading bacteria, indicates the increase of dangerous signals in the rumen epithelium of the HC group. Overall, our results suggest that the regulatory effects of ruminal SCFAs on cellular activities of physical barrier play important roles in the modulation of immune responses in the rumen epithelium.

Totally, this study suggested that the medium increase of ruminal SCFAs promotes the diversity of epimural microbiota and its gene pool. Meantime, it induced the commensal lifestyle of opportunistic pathogens in the epimural microbiota. Furthermore, the moderate concentration of ruminal SCFAs suppressed the inappropriate immune responses, and promoted the tight junction and cell refreshing in the rumen epithelium. However, the further increase of ruminal SCFAs resulted in the acidification of rumen, leading to a decrease in the diversity of epimural microbiota and its gene pool. Meantime, it induced the pathogenic lifestyle of opportunistic pathogens in the epimural microbiota. Additionally, the acid environment in the rumen led to the upregulation of immune responses, the overgrowth of the epithelial cells, and the downregulation of tight junction and gap junction pathways in the rumen epithelium. Altogether, our study indicated a simultaneous regulation of ruminal SCFAs on the microbial, physical and immune barriers by their concentrations and the impacts on the ruminal pH.

## Ethics Statement

This study was carried out in accordance with the recommendation of the Regulations for the Administration of Affairs Concerning Experimental Animals (No. 588 Document of the State Council of the People’s Republic of China, 2011). The protocol was approved by the Nanjing Agricultural University.

## Author Contributions

HS wrote the manuscript. HS and ZX analyzed the data. ZL and ZS designed the research. ZL performed the experiments. All authors approved the final manuscript.

## Conflict of Interest

The authors declare that the research was conducted in the absence of any commercial or financial relationships that could be construed as a potential conflict of interest.

## Supplementary Material

FIGURE S1 Effects of LC, MC, and HC diets on the diversity of epimural microbiota.

FIGURE S2 Linear regression analysis of relationships between RNA-seq results and RT-QPCR results for 11 selected genes.

TABLE S1 Primers used for the RT-QPCR.

TABLE S2 Genera composition and abundances of epimural microbiota.

TABLE S3 KO compositions and abundances of epimural microbiota.

TABLE S4 Gene compositions and abundances in the rumen epithelium.

TABLE S5 Differentially expressed genes in the pathways related to the immune system and cellular processes when the diets shifted from LC to MC.

TABLE S6 Differentially expressed genes in the pathways related to the immune system and cellular processes when the diets shifted from MC to HC.

TABLE S7 RT-QPCR results of 11 selected genes that were differentially expressed in the rumen epithelium among groups.
