# Supplementary figures and images for: The Regulation of Ruminal Short-Chain Fatty Acids on the Functions of Rumen Barriers
Source: Front Physiol. 2019 Oct 25;10:1305. doi: 10.3389/fphys.2019.01305 (PMC6842973; doi:10.3389/fphys.2019.01305)

Fig. S1 Effects of LC, MC, and HC diets on the diversity of epimural microbiota

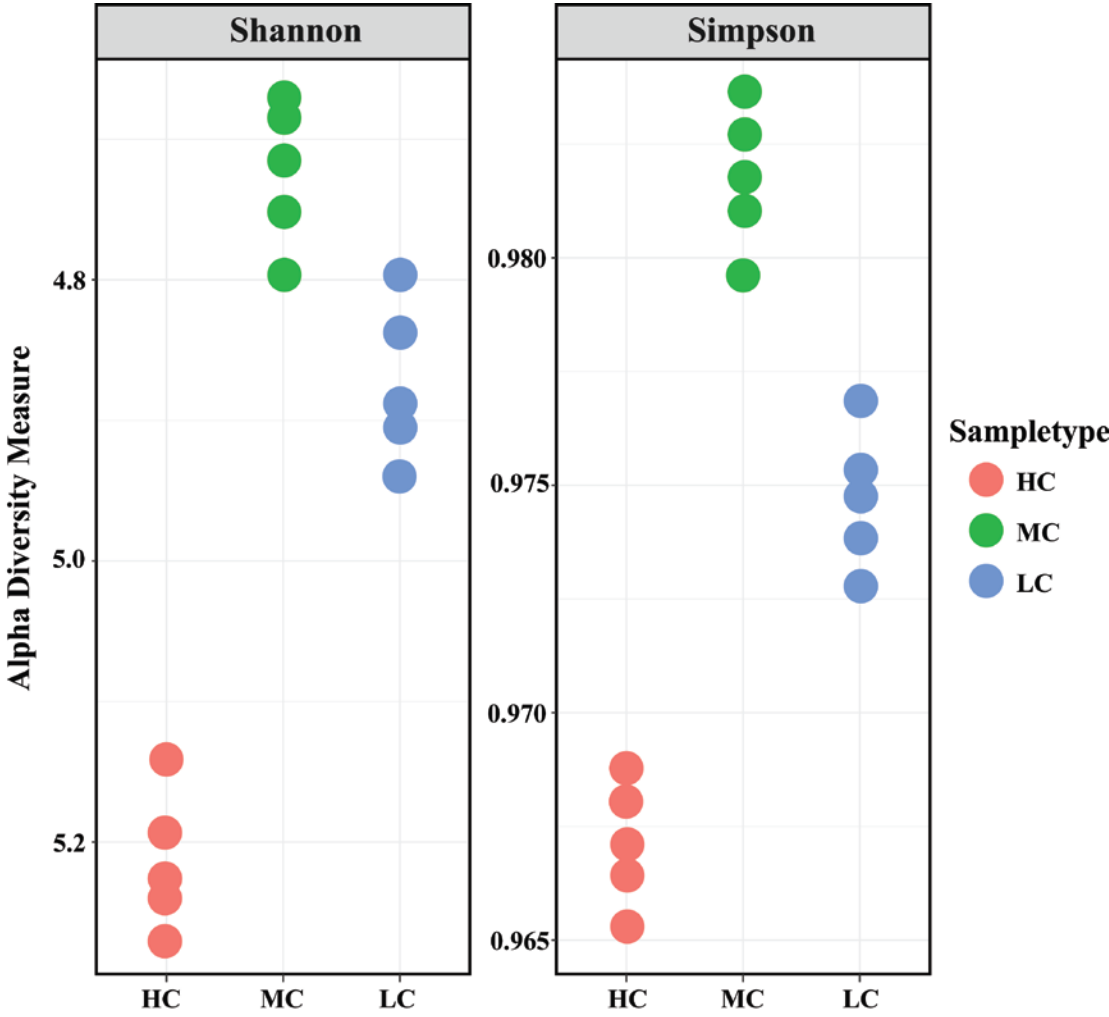

Supplement: FIGURE S1 — Effects of LC, MC, and HC diets on the diversity of epimural microbiota. [file Data_Sheet_1.PDF]
