# Supplementary material for: The Regulation of Ruminal Short-Chain Fatty Acids on the Functions of Rumen Barriers
Source: Front Physiol. 2019 Oct 25;10:1305. doi: 10.3389/fphys.2019.01305 (PMC6842973; doi:10.3389/fphys.2019.01305)

Fig. S2 Linear regression analysis of relationships between RNA-seq results and RT-QPCR results for 11 selected genes.

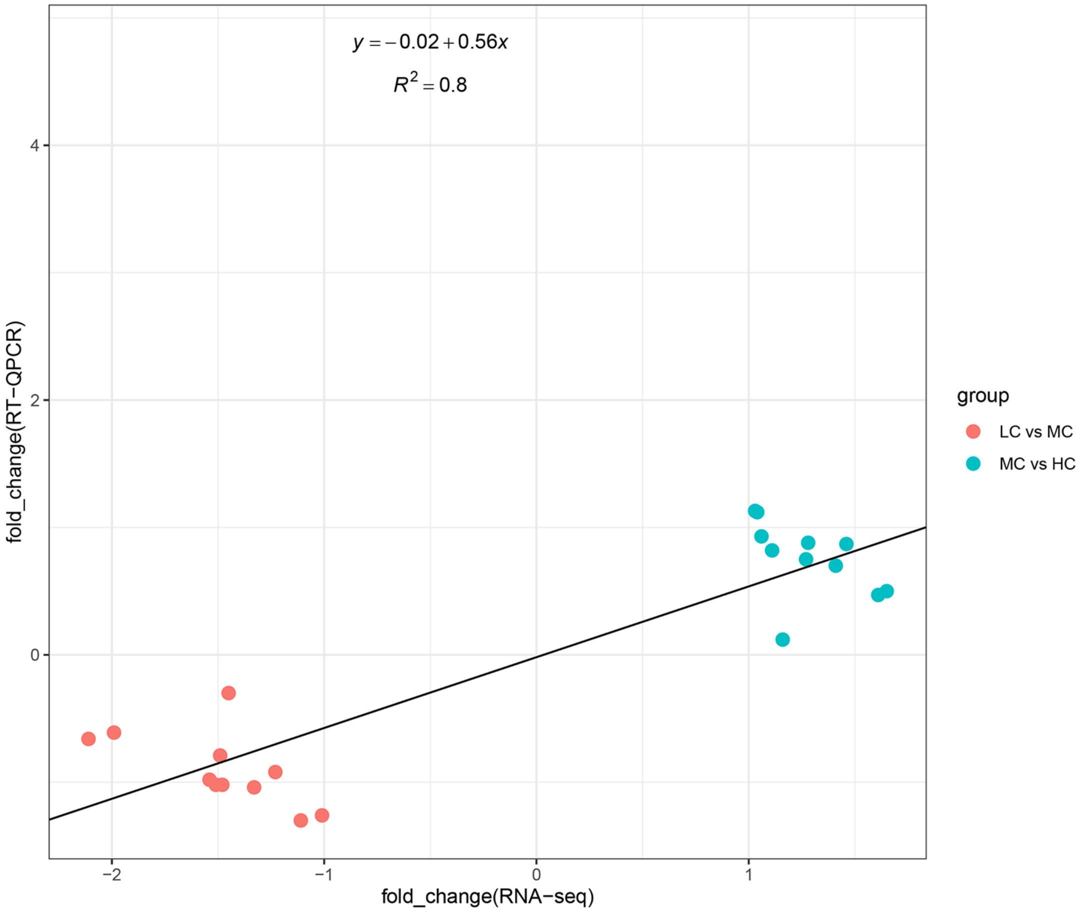

Supplement: FIGURE S2 — Linear regression analysis of relationships between RNA-seq results and RT-QPCR results for 11 selected genes. [file Data_Sheet_2.PDF]
